# Cytomegalovirus pneumonia presenting as pulmonary nodules

**DOI:** 10.4322/acr.2021.362

**Published:** 2022-03-09

**Authors:** Janet Basinger, Meghan E. Kapp

**Affiliations:** 1 Vanderbilt University Medical Center, Department of Pathology, Microbiology, and Immunology, Nashville, Tennessee, United States

**Keywords:** Autopsy, Cytomegalovirus, Multiple Pulmonary Nodules, Pneumonia, Transplant Recipients

## Abstract

Cytomegalovirus (CMV) pneumonia is a well-known cause of morbidity and mortality in patients with a history of allogenic hematopoietic stem cell transplant. Radiographically, CMV pneumonia most commonly presents as bilateral ground glass opacities; however, the presentation is non-specific and can be variable, including presenting as areas of air-space consolidation or pulmonary nodules. We report a case of a 70-year-old man who presented with rapidly progressive bilateral pulmonary nodules approximately two months after receiving a bone marrow transplant. No infectious etiology was identified for the pulmonary nodules, and a bronchoscopy was unable to be performed due to a rapid decline in the patient’s overall condition and respiratory status. The patient died shortly after the decision was made to transition to palliative care and a limited autopsy was performed to explore the pulmonary findings. Corresponding to premortem imaging were the postmortem gross findings of numerous bilateral pulmonary nodules and a large mass-like area of consolidation in the right upper lobe. Microscopic examination of the nodules demonstrated a necrotizing pneumonia with few foci of viral cytopathologic change consistent with CMV, which was confirmed by immunohistochemistry. While CMV is a common infectious agent in the immunocompromised population, CMV pneumonia continues to be a challenging entity due to difficulty in diagnosis and treatment. Rapidly enlarging pulmonary nodules in an immunosuppressed patient is highly suggestive of an infectious process and careful histologic examination for viral cytopathologic change is essential.

## CASE REPORT

**Figure 1:**
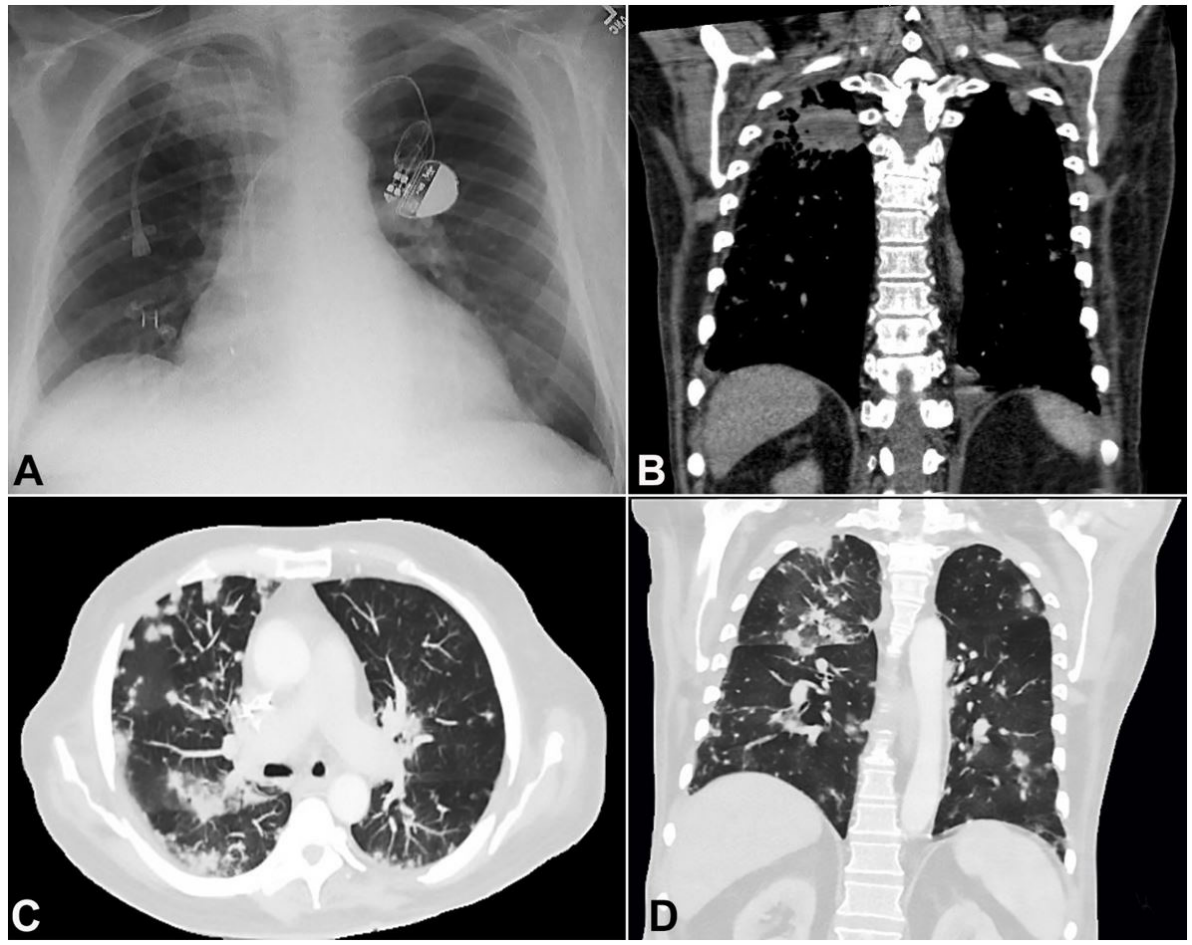
**A** – Initial Chest X-ray, PA view, Ill-defined right apical soft tissue density with relative volume loss of right hemithorax; **B**, **C**, and **D** – Follow-up Chest CT scan, sagittal view, multiple poorly marginated nodules throughout the lungs ranging in size up to 1.5 cm in the left upper lobe and partial clearance of prior right upper lobe pneumonia (**B** coronal plane – mediastinum window, **C** – axial plane lung window, **D** coronal plane lung window).

A 70-year-old man with a complicated past medical history including pacemaker dependent atrial fibrillation, severe osteoarthritis, and acute myeloid leukemia arising from chronic myelomonocytic leukemia presented to the hospital at day 55 status post allogenic stem cell transplant with severe joint pain, weakness, anorexia, and failure to thrive. His hospital course was complicated by multiple viral infections, including BK virus cystitis, Epstein Barr Virus (EBV) reactivation, and Cytomegalovirus (CMV) viremia. He had been admitted to the hospital one-month prior with a right upper lobe pneumonia evidenced by a right upper lobe consolidation on chest x-ray (Figure 1A), which was confirmed by chest computed tomography (CT). A follow up chest CT four weeks later showed partial clearance of the right upper lobe consolidation in addition to extensive involvement of all lobes of the lungs by new poorly marginated nodules in the peripheral and central portions of the lungs, measuring up to 1.5 cm in greatest dimension with suggestions of central cavitation (Figure 1B and 1C).

**Figure 2:**
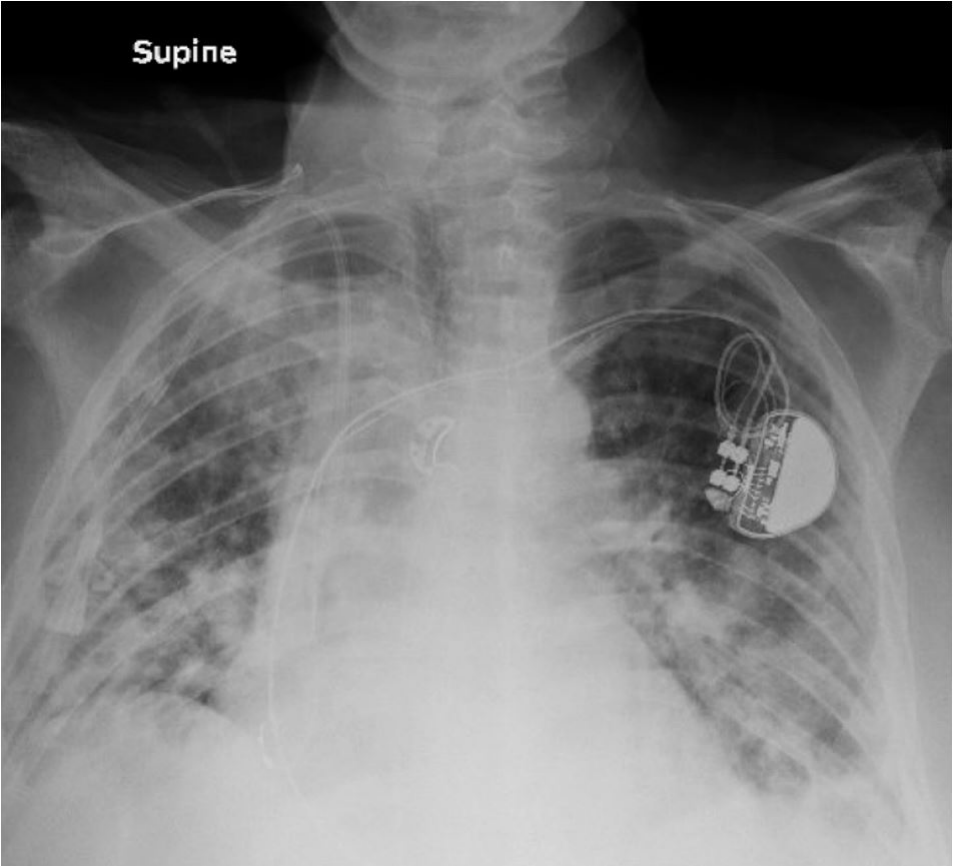
Subsequent Chest X-ray, AP view, Significant increase in nodular airspace opacities bilaterally with associated small bilateral pleural effusions and persistent consolidation in the right upper lobe.

On a chest X-ray from the current hospital admission, the right upper lobe consolidation was persistent, and the nodularity had significantly increased bilaterally (Figure 2).

Additional infectious disease evaluation for pneumonia was negative, and empiric antifungal agents were administered. A bronchoscopy was scheduled; however, the patient experienced a rapid decline and worsening respiratory status, and the decision was made to transition to comfort care measures. The patient died shortly after and consent was obtained for postmortem examination limited to the lungs.

## AUTOPSY FINDINGS

**Figure 3:**
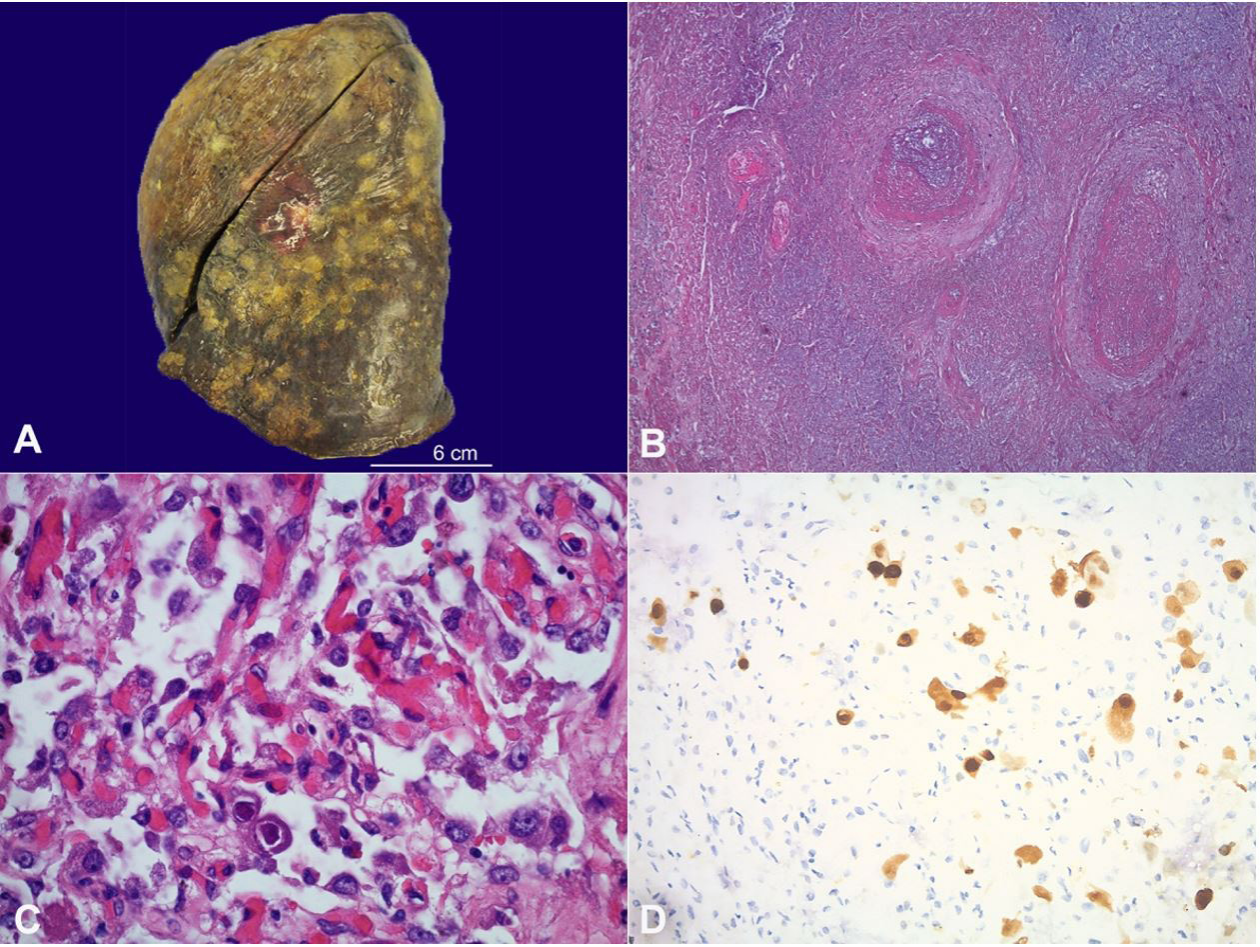
**A** – Left lung, post-fixation, Scattered subpleural tan-white, variably sized nodules; **B** – Lung nodule, necrotizing pneumonia, involving the bronchovascular bundles and lung parenchyma (H&E 40x); **C** – Lung, Type II pneumocytes with viral cytopathologic change, consistent with cytomegalovirus (H&E 400x); **D** – Lung, CMV IHC, Strong nuclear positive staining in CMV infected cells (400x).

Postmortem examination of the lungs revealed numerous pleural and parenchymal nodules (Figure 3A) present in all lobes of the lungs, of which some are necrotizing. A 6 cm mass-like consolidation in the right upper lobe represented the prior pneumonia. There were small pleural effusions bilaterally. The bronchial mucosa was tan-white and glistening without evidence of bronchitis or mucous plugging. There were no pulmonary emboli present. Microscopic examination of the nodules revealed necrotizing pneumonia (Figure 3B) with associated diffuse alveolar damage and foci of organizing pneumonia. There were few scattered cells demonstrating cytomegaly and large, basophilic intranuclear inclusions with a peripheral halo (Figure 3C) consistent with CMV confirmed by immunohistochemistry (IHC) (Figure 3D). Additional studies for HSV-1/2, gram stain, GMS, and AFB were negative. The immediate cause of death was determined to be CMV pneumonia involving all lobes of the lungs with associated diffuse alveolar damage.

## DISCUSSION

Cytomegalovirus is a common opportunistic infection, and a well-documented infectious agent in patients with a history of hematopoietic stem cell transplant (HSCT). The number of CMV pneumonia related deaths has declined in this population over the years with advancements in viral serology testing and prophylactic and therapeutic antiviral therapy.^1^^,^ 2 However, there remains diagnostic and treatment challenges, with mortality rates over 85% with delayed treatment or ineffective prophylaxis.^3^

The diagnosis of CMV pneumonia is based on either lung tissue culture, bronchoalveolar lavage, or histologic examination of lung tissue with concurrent clinical or radiographic evidence of pneumonia.^1^^,^ 3 Autopsy-proven CMV pneumonia is characterized by the presence of CMV-type viral cytopathologic change, although tissue culture for CMV has also been used.^2^ The radiographic findings are variable, and while the most common presentation is reported as ground glass opacities, air space consolidation and pulmonary nodules are also reported.^4^ These findings are not specific for CMV pneumonia as pulmonary nodules can be the presentation for many infectious entities including other viral pathogens (e.g. Varicella-Zoster Virus), fungal pathogens (e.g. Candida species, Cryptococcus), and miliary tuberculosis, among others.^5^ Additionally, neoplasia must be considered and excluded in the setting of pulmonary nodules, although in the setting of a rapidly progressive and diffuse process this is much less likely.

Pneumonia in HSCT patients can be divided by whether it occurs early (30-days post-engraftment) or late (greater than 30-days post-engraftment) after transplantation. It is reported that 25% of patients who have received a HSCT go on to develop at least one episode of pneumonia late after transplantation, with the most common etiology being viral.^6^ In one study, CMV was the most common virus identified in these cases, accounting for 22 out of the 33 late viral pneumonias and continues to be associated with high mortality rates. Erard et al.^1^ reported 87% of patients who developed CMV pneumonia died within 25 days from onset of the disease. Early initiation of appropriate therapy is critical in these cases, as delayed therapy in patients who experience respiratory failure requiring mechanical ventilation is reported to be 100%.^3^ Additionally, CMV viremia is not necessarily a predictor of CMV disease as it may or may not precede tissue-invasive disease. It is an indication for initiation of antiviral therapy as CMV viremia is an independent risk factor for increased mortality in the first year after HSCT.^7^^,^ 8

The gross findings of CMV pneumonia are nonspecific and variable, as is seen with the radiographic findings. If concurrent diffuse alveolar damage is present microscopically, the gross findings associated with DAD may also be appreciated, but these are also non-specific. In this case, there was a large mass-like area of consolidation as well as scattered peripheral and central pulmonary nodules, a subset of which were necrotizing, which may raise suspicion for lung cancer, but the rapid clinical presentation makes this unlikely.^9^ The microscopic findings of CMV are classically described as cytomegaly with large, basophilic inclusions with a peripheral halo, forming the characteristic “owl’s eye” nucleus. Also, smaller intracytoplasmic granular inclusions may also be seen. These findings; however, are not entirely specific, as there may be overlap with viral cytopathologic changes associated with other viruses. The use of IHC should be considered on a case-by-case basis, especially in cases with extensive necrosis, minimal or questionable viral cytopathologic change, or concern for multiorganism disease.^10^ Tissue culture and additional ancillary studies, such as tissue quantitative nucleic acid amplification, may be more sensitive however there is the possibility of contamination in cases of CMV viremia, so the gold standard for diagnosis of tissue-invasive CMV disease is histopathologic diagnosis with or without IHC confirmation.^11^

## CONCLUSION

Cytomegalovirus pneumonia is a common entity in hematopoietic stem cell transplant patients. The radiographic findings are not specific and may present as pulmonary nodules, which can be seen in a number of infectious and neoplastic processes. CMV pneumonia in the late transplantation period is associated with a high mortality rate. The prophylactic and presumptive treatment measures are effective, but mortality rates remain high. The gross autopsy findings can be variable and include consolidation, mass-like lesions, and pulmonary nodules, all of which were present in this case. Tissue must be carefully assessed for CMV viral cytopathologic changes in these autopsy cases. The utilization of immunohistochemistry and molecular studies as adjuncts to histologic examination should be determined on a case-by-case basis, but may be particularly useful in cases with minimal viral cytopathologic changes or extensive necrosis. The mortality rate associated with late stage CMV pneumonia remains high due to continued difficulty with diagnosis and effective treatment.

## References

[1] Erard V, Guthrie KA, Seo S (2015). Reduced mortality of cytomegalovirus pneumonia after hematopoietic cell transplantation due to antiviral therapy and changes in transplantation practices. Clin Infect Dis.

[2] Torres HA, Aguilera E, Safdar A (2008). Fatal cytomegalovirus pneumonia in patients with haematological malignancies: an autopsy-based case–control study. Clin Microbiol Infect.

[3] Nguyen Q, Champlin R, Giralt S (1999). Late cytomegalovirus pneumonia in adult allogeneic blood and marrow transplant recipients. Clin Infect Dis.

[4] Gasparetto EL, Ono SE, Escuissato D (2004). Cytomegalovirus pneumonia after bone marrow transplantation: high resolution CT findings. Br J Radiol.

[5] Shimada A, Koga T, Shimada M (2004). Cytomegalovirus pneumonitis presenting small nodular opacities. Intern Med.

[6] Chen CS, Boeckh M, Seidel K (2003). Incidence, risk factors, and mortality from pneumonia developing late after hematopoietic stem cell transplantation. Bone Marrow Transplant.

[7] Meyers JD, Ljungman P, Fisher LD (1990). Cytomegalovirus excretion as a predictor of cytomegalovirus disease after marrow transplantation: importance of cytomegalovirus viremia. J Infect Dis.

[8] Green ML, Leisenring W, Xie H (2016). Cytomegalovirus viral load and mortality after haemopoietic stem cell transplantation in the era of pre-emptive therapy: a retrospective cohort study. Lancet Haematol.

[9] Karakelides H, Aubry MC, Ryu JH (2003). Cytomegalovirus pneumonia mimicking lung cancer in an immunocompetent host. Mayo Clin Proc.

[10] Solomon IH, Hornick JL, Laga AC (2017). Immunohistochemistry is rarely justified for the diagnosis of viral infections. Am J Clin Pathol.

[11] Kotton CN, Kumar D, Caliendo AM (2018). The third international consensus guidelines on the management of cytomegalovirus in solid-organ transplantation. Transplantation.

